# Microvascular flow dictates the compromise between spatial resolution and acquisition time in Ultrasound Localization Microscopy

**DOI:** 10.1038/s41598-018-38349-x

**Published:** 2019-02-21

**Authors:** Vincent Hingot, Claudia Errico, Baptiste Heiles, Line Rahal, Mickael Tanter, Olivier Couture

**Affiliations:** Institut Langevin, CNRS, INSERM, ESPCI Paris, PSL Research University, 17 rue Moreau, 75012 Paris, France

## Abstract

Medical ultrasound is a widely used diagnostic imaging technique for tissues and blood vessels. However, its spatial resolution is limited to a sub-millimeter scale. Ultrasound Localization Microscopy was recently introduced to overcome this limit and relies on subwavelength localization and tracking of microbubbles injected in the blood circulation. Yet, as microbubbles follow blood flow, long acquisition time are required to detect them in the smallest vessels, leading to long reconstruction of the microvasculature. The objective of this work is to understand how blood flow limits acquisition time. We studied the reconstruction of a coronal slice of a rat’s brain during a continuous microbubble injection close to clinical concentrations. After acquiring 192000 frames over 4 minutes, we find that the biggest vessels can be reconstructed in seconds but that it would take tens of minutes to map the entire capillary network. Moreover, the appropriate characterization of flow profiles based on microbubble velocity within vessels is bound by even more stringent temporal limitations. As we use simple blood flow models to characterize its impact on reconstruction time, we foresee that these results and methods can be adapted to determine adequate microbubble injections and acquisition times in clinical and preclinical practice.

## Introduction

With a sub-millimeter precision deep in tissues, medical ultrasound is a widespread preclinical and clinical technique to study organs and blood flows. However, its spatial resolution is restricted by diffraction to about a half-wavelength and penetration is reduced for shorter wavelengths. Thus, direct imaging of deep microscopic structures and particularly the microvascular network is impossible. This limitation blinds ultrasound to the microvasculature that is a key aspect of major diseases such as cancer, stroke, diabetes and arteriosclerosis.

Recently, super-resolution techniques, such as fPALM (fluorescence Photo Activated Localization Microscopy^1^), showed the way to overcome the diffraction limit in optical microscopy. It relies on the localization of individual punctual fluorescent sources which are separated, frame-by-frame, through their stochastic activation. Accumulating their sub-wavelength localizations over thousands of acquisitions allow the formation of an image with a resolution improved more than ten times. Ultrasound Localization Microscopy (ULM) was developed as a transposition of fPALM to ultrasound imaging^2–15^ using clinically-approved gaseous contrast agents, smaller than red blood cells. After intravenous injection, these microbubbles, explore the whole vasculature and are eliminated within a few minutes. As in fPALM, the key to super-resolution is the imaging of isolated echoes from individual sources. For ULM, that means isolating individual microbubbles signatures from surrounding tissues, blood and other microbubbles. Microbubbles can be distinguished through their nonlinear echoes, at conventional^7–9^ or ultrafast^10–12^ frame rates, or through their motions using Singular Value Decomposition (SVD) filters^3,14–16^. In all these methods, at most tens of microbubbles can be separated in each frames. The precise positions of hundreds of thousands of individual microbubbles are then accumulated to form super-resolution images. Under these conditions, ULM was able to map vessels smaller than 10 μm with velocities as low as 1 mm.s^−1^ in the living rat brain^2,3^ and image the microvasculature of the kidney^10,12^, ear^9^ and tumor in animal models^11,13^. Despite these promising results, ULM faces limitations that need to be understood. The most important are the long acquisition times required to form a complete image. For example, 3 minutes was required to map the rat’s brain vasculature at an 8 microns resolution^3^.

Fundamentally, for a vessel to be reconstructed, a least one microbubble has to be unambiguously detected, localized and tracked in it. However, the number of microbubbles injected^17^ (~10^8^) is dominated by the total number of red blood cells^18^ (~10^13^) which leads to very rare microbubbles passage in the vessels with the slowest flow^19^ (<1 mm.s^−1^). This means that the reconstruction time for the smallest vessels is above all determined by the slowest flows.

In this study, we explore the link between the slow passage of microbubbles inside the vasculature and the acquisition time for Ultrasound Localization Microscopy. In the next section, we introduce a model Equation [4] based on blood flow to describe how it drives acquisition time and spatial resolution. We then apply it to the rat’s brain as it offers a large and multiscale distribution of vessels. We introduce different analysis methods to study image formation and vessel reconstruction. We first use saturation methods to describe general image reconstruction and propose a general inverse relation between acquisition time and pixel size. We then use a local approach on individualized vessels to investigate how blood flow affects a vessel’s reconstruction, especially the slowest ones. We finally describe the effect of acquisition time on the determination of velocity profiles. Finally, we discuss the validity and relevance of this framework and especially how it can serve as a guideline for ULM acquisitions in 2D and eventually 3D, and to accompany the translation to clinical practice.

## Theory

Blood flow in the microvasculature displays various complex behaviors^20^, mainly pulsatility in small arteries (1–5 mm in diameter), the contractility of lean muscles in the arterioles (20–200 μm), and rheology in capillaries (a few μm). However, blood flow in vessels *Q* follows an overall Poiseuille law^20^ [1], with *L* corresponding to vessel’s length, *η* to the blood viscosity and ΔP to the continous pressure drop between small arteries (90 mmHg) and big veins (10 mmHg). The most important parameter however is the diameter (*d)* as suggested by the exponent 4. ULM can accurately predict vessels’ sizes and average velocities but lacks information on pulsatility, contractility and rheology. A reductionist approach to blood flow using only measurable ULM parameters is to set vessel’s diameter as the main variable even though it misses a lot of hemodynamics effects and poorly describes blood flow.

1 $$Q=\frac{{\rm{\pi }}{d}^{4}}{128\eta }\frac{{\rm{\Delta }}{\rm{P}}}{L} \sim Q(d)$$

We also assume that a minute after the beginning of microbubble infusion, microbubbles are evenly distributed in the blood circulation. Therefore, we can count the microbubbles passing through a vessel as a measure of the blood flow. In other words, the average number of microbubbles N crossing a section of a vessel of diameter *d* after time *T*_*acq*_ while having a vascular concentration *C*_*bubbles*_ is given by Equation [2].

2 $$N=Q(d)\ast {C}_{bubbles}\ast {T}_{acq}$$

In most vessels, blood flows mainly in only one preferential direction. Continuous imaging with high framerates (>500 Hz) allows accurate tracking of the microbubbles for several tens of milliseconds. Therefore, a continuous trace can be reconstructed from one individual microbubble reducing the dimensionality of the problem by an order of magnitude as a surface is reconstructed with lines. The number of pixels *N* necessary to reconstruct a vessel of diameter *d* with pixels of size *l*_*pix*_ is given by Equation [3].

3 $$N=\frac{d}{{l}_{pix}}$$

Considering that the reconstruction of a single vessel requires at least one microbubble detection per pixel, [2] and [3] give [4] and determines the minimum acquisition time *T*_*acq*_ needed to reconstruct a vessel of diameter *d* with a pixel size *l*_*pix*_ and a vascular concentration of microbubbles *C*_*bubbles*_.

4 $${T}_{acq}=\frac{1}{\frac{Q(d)}{d}{C}_{bubbles}{l}_{pix}}$$

## Materials and Methods

### Animal procedure

The signal to noise ratio is a critical factor in the localization of the microbubbles^6^. To study the kinetics of ULM close to the theoretical resolution limit, the skull bone had to be removed to maximize the contrast between microbubbles and tissues and to limit aberrations. As in previous studies^3,15^, Adult Sprague Dawley rats were anesthetized with medetomidine (Domitor^®^, 0.3 mg.kg^−1^) and ketamine (Imalgène^®^, 40 mg.kg^−1^), a catheter was placed in the jugular vein and the head of the animal was fixed in a stereotaxic frame. A cranial window was carved using a 1.4 mm drill and the skull bone was removed, leaving the brain intact. Agar gel was put on the brain to make a protecting layer and ultrasound gel was put to ensure coupling with the probe. Vital parameters (body temperature, breathing frequency) were checked regularly to ensure stability of the animal. All procedures were performed in accordance with the European Community Council Directive of 22nd September 2010 (010/63/UE) and approved by the institutional committee C2EA-59: “Comité d'éthique en matière d’expérimentation animale Paris Centre et Sud” under the protocol 2015–23.All experiments were performed in accordance with the ARRIVE guidelines.

### Microbubble injection

400 μL of Sonovue microbubbles were injected through a catheter placed in the jugular vein at a steady rate of 80 μL.min^−1^ for 5 minutes to keep a stable concentration in the blood stream. After 1 minute of injection, perfusion of microbubbles in the vasculature reached a steady state and acquisition was started. The rats weigh 500 g which correspond to microbubble injections of 0.8 ml.kg^−1^, higher than clinical recommendations^21^ (0.03 ml.kg^−1^) but below safety tested doses^22^ (<1 ml.kg^−1^) for Sonovue. After image processing, we found an average of 30 microbubbles per frame (or 24 000 microbubbles every second) with a standard deviation of 5% along the different blocks.

### Ultrafast ultrasound acquisition

240 blocks of 800 compounded frames, with angles at −5°, 0°, +5°, at 1000 Hz at a depth of 10 mm were acquired every second using a 15 MHz probe (Vermon, Tours, France) with a pitch of 0.1 mm which enables a 100 μm × 100 μm in plane resolution. The elevation focusing is done by a plastic lens to reach 500 μm at 8 mm in depth. Acquisitions were performed on a programmable ultrafast ultrasound scanner (SuperSonic Imagine, Aix-En-Provence, France).

### ULM Image processing

Small rigid motions due to cerebral pulsatility were corrected through phase-correlation^15^. As described in previous studies^2,3^, Singular Value Decomposition filters^16^ were used to extract a microbubble’s signal from surrounding tissues by removing only the two first singular values and applying a second order Butterworth high pass filter with a cutoff frequency of 20 Hz to remove remaining tissue signal. Microbubbles were localized using a method adapted from optical microscopy^23^: microbubbles are detected as the brightest local maxima on the image. Then, the position of the microbubbles within the pixel is calculated using a weighted average on the intensity in the neighboring pixels. Microbubbles tracks were formed by pairing microbubbles between frames using a tracking algorithm (simpletracker, Mathworks). It is based on the Hungarian method for assignment^24^. For a microbubble, distances with all the microbubbles in the following frame are calculated. The Kuhn-Munkres algorithm then minimizes the total distance thus connecting all the positions of a microbubble to form a collection of its position in the different frames. Velocities are calculated as the mean displacement between two consecutive frames. The microbubble track is completed using linear interpolation to fill missing points and smoothed using a sliding average along 5 positions. Finally the positions are rounded to the chosen pixel sizes and a density image can be displayed as the projection of all microbubbles tracks, a velocity image can be reconstructed as the mean velocity of all projected microbubbles. Detailed information on image processing for ULM can be found in a recent review article^2^.

### Saturation methods

We assessed the reconstruction of the image as the number of reconstructed pixels. For pixel sizes between 5 μm and 100 μm, partial images were reconstructed with only fractions of the dataset corresponding to different accumulation times. Counting the number of explored pixels at a given time point enables the determination of saturation curves. As these curves exhibit standard saturation behavior, a measure of the characteristic time involved is the slope of the saturation curve at its origin. For each pixel size, we estimated a characteristic time T_res_ as the intersection between the tangent at origin and the 100% saturation line.

### Vessel individualization and simplification of the model equation

For local quantification, the cross sections of 150 vessels with diameters ranging between 5 μm and 90 μm were manually delimited. Crossing microbubbles were counted and velocity profiles could be reconstructed. Local saturation curves can be calculated to determine reconstruction time of each vessel. To simplify the link between acquisition time and vessel diameter in [4], we introduce the variable *h(d)* in [5] that can be calculated with *d* and *N(d)*. Therefore, we can represent the influence of the diameter with only one variable *h*. With [2], we can also write *h(d)* as [5′].

5 $$h(d)=\frac{N(d)}{d{T}_{acq}}$$

and

5&x2032; $$h(d)=\frac{Q(d)}{d}{C}_{bubbles}$$

The acquisition time determined in [4] can then be simplified in [6]. Equivalence between *h* and the underlying vessel diameter is displayed on top of the corresponding curves for more clarity.

6 $${T}_{acq}=\frac{1}{\frac{Q(d)}{d}{C}_{bubbles}{l}_{pix}}=\frac{1}{h(d)\,{l}_{pix}}$$

### Two distinct measures of blood flow

As we analyze microbubbles flowing in a vessel, we can use two different approaches to estimate blood flow. A mesoscopic approach consists in counting microbubbles passing through a given cross section [7]. A microscopic approach consists in measuring microbubbles velocities and the vessel diameter. The flow can be written using the average velocity [7′].

7 $$Q(d)=\frac{N(d)}{{C}_{bubbles}{T}_{acq}}$$

and

7&x02032; $$Q(d)={v}_{mean}(d)\pi {d}^{2}$$

### Estimation of microbubble concentration in the blood

Using the two estimations of the blood flow [7] & [7′], we can average these measure on the 150 vessels individually segmented and estimate the actual concentration in the cerebral blood of the rat [8].

8 $${C}_{bubbles}=mean(\frac{N(d)}{v(d)\pi {d}^{2}{T}_{acq}})\sim {2.10}^{5}\,MB.m{l}^{-1}$$

## Results

### Microbubbles can be localized and tracked with 5 μm accuracy

**Figure 1:**
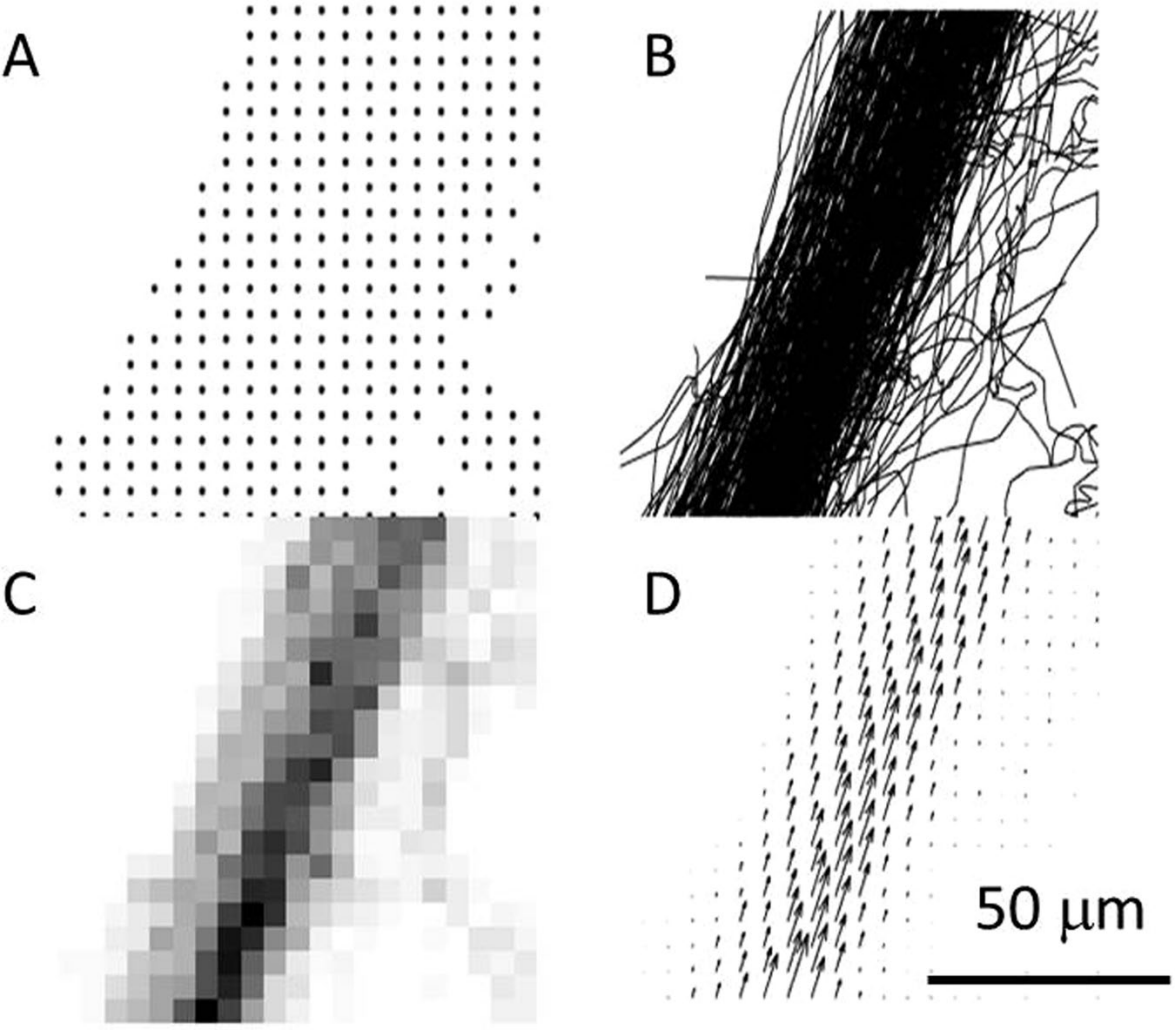
Different modalities of ULM in a 50 μm vessel reconstructed with 5 μm pixels (interpolation factor 15) after 240 s. (**A**) All microbubbles detected positions. (**B**) Corresponding microbubbles tracks. (**C**) Map of density count of microbubbles. (**D**) Average velocity field.

In the course of 4 minutes, 192,000 coronal planes of an *in vivo* rat brain were acquired with an ultrafast ultrasound scanner during a constant jugular infusion of contrast agents. 6 million microbubbles were localized with interpolation factors ranging from 1 to 15 (Fig. 1A), corresponding to in plane pixel sizes between 100 μm and 5 μm which is the theoretical resolution limit described in (4). Tracking individual microbubbles throughout consecutive frames was done to form continuous tracks (Fig. 1B). Density maps can be reconstructed by counting the number of microbubbles detected in each pixel (Fig. 1C). Instantaneous velocity along the tracks is calculated and an average velocity field can be reconstructed (Fig. 1D).

### ULM image can be reconstructed with 5 μm pixels

**Figure 2:**
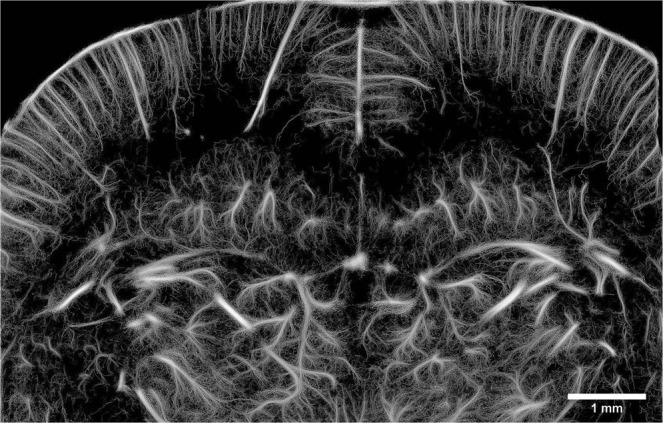
ULM of a coronal slice of the rat’s brain reconstructed with 5 μm pixels after 240 s of acquisition.

After 4 minutes of acquisition and about 6 million microbubbles detected, a coronal plane can be reconstructed with pixels of 5 μm (Fig. 2) which is the precision limit for a microbubble’s localization as determined in [4].

### Global approach: Influence of acquisition time on the reconstruction pixel size

**Figure 3:**
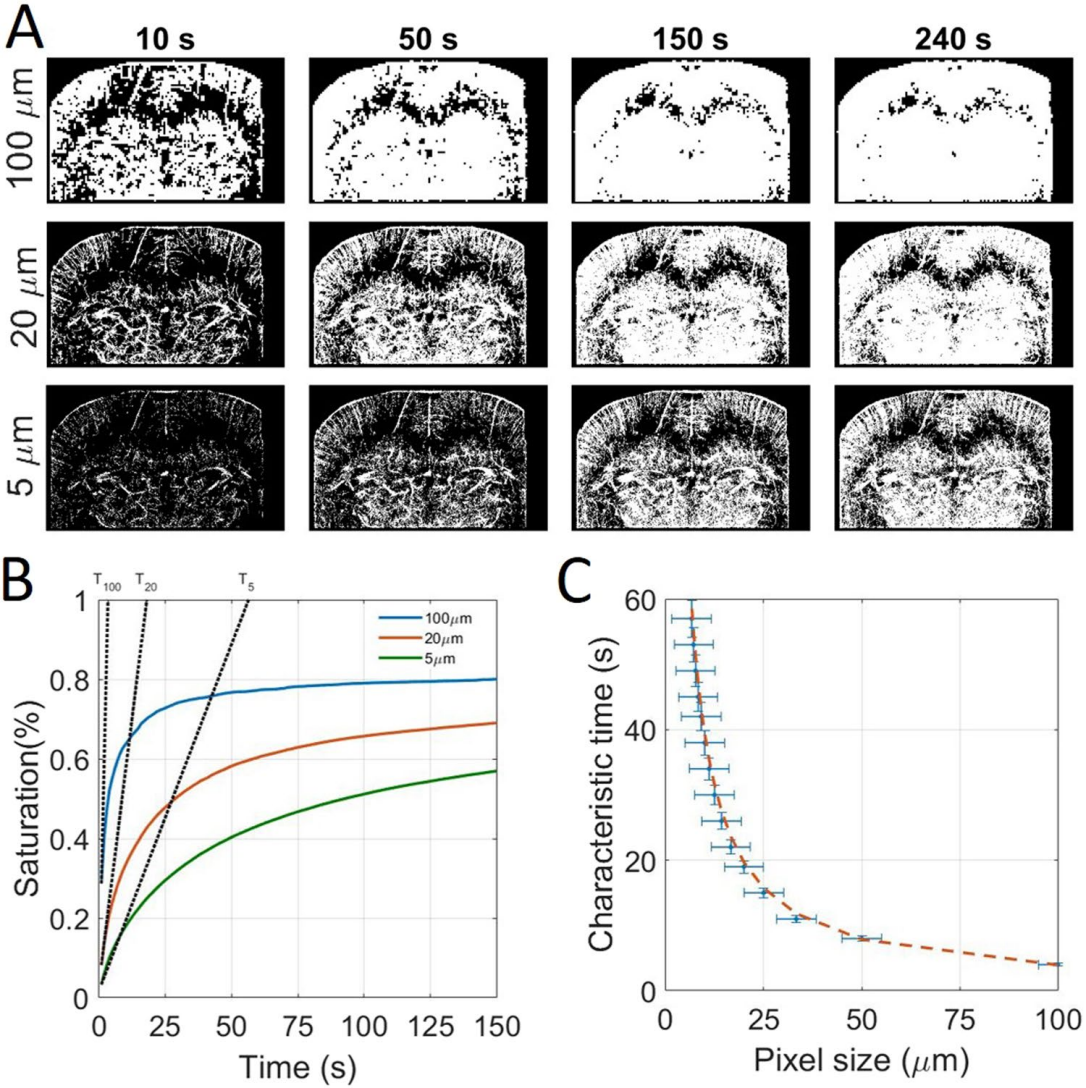
Global approach using whole image saturation and determination of the influence of the acquisition time on reconstruction pixel size. (**A**) Saturated Images for different pixel sizes and after different acquisition times. (**B**) Corresponding saturating curves with the tangent at origin intersecting the 100% saturation line to determine the saturation time. (**C**) Experimental time constants as a function of the pixel size and inverse fit ${y}={{p}}_{1}\frac{1}{{\boldsymbol{x}}}$ with p_1_=384 s. μm^−1^, R^2^ = 0.99.

To study the tradeoff between spatial and temporal resolution, we reconstructed images with different pixel sizes (between 5 μm and 100 μm) and after different accumulation times (between 1 s and 240 s). Reconstructed pixels were saturated after the accumulation of a single microbubble to make binary images as shown in Fig. 3A. Saturation curves in Fig. 3B show the temporal behavior of image reconstruction. After a rapid phase, the reconstruction slows down as microbubbles are filling the microvascular bed. If for the images with 100 μm pixels the saturation curve reached a plateau within the time of the experiment, the corresponding 5 μm pixel image did not reach saturation. However, we postulate that a reasonable image quality is reached at 90% of final saturation, with a good sampling of micro vessels being displayed in the image. From the saturation curve with 100 μm pixels, which is fully saturated, we see that 90% saturation is reached after about 3 times the characteristic time. We extrapolate this behavior to estimate a reconstruction time as 3 times the characteristic time. Consequently, an image with 100 μm pixels would be reconstructed within 10 s. This reconstruction time increases to 60 s for 20 μm pixels and gets close to 4 minutes for 5 µm pixels.

### Local approach: Influence of acquisition time on the vessel reconstruction

**Figure 4:**
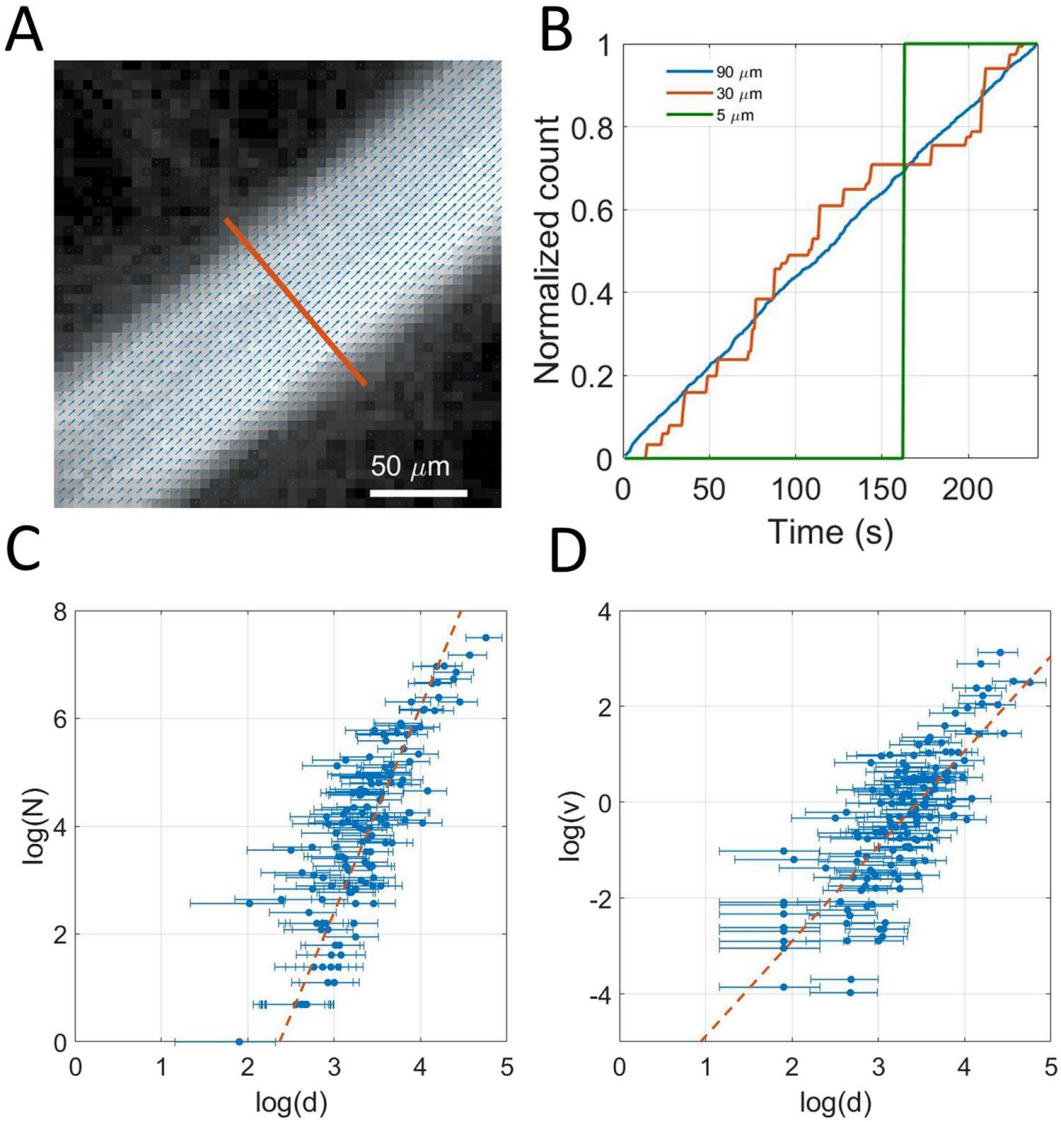
Local approach using 150 individualized vessels. (**A**) Example of a selected vessel’s section superimposed on density image with velocity field (**B**). Example of time dependency of microbubbles count in three vessels of 80 μm, 20 μm and 5 μm. (**C**) Dependency of the bubble rate with vessel’s diameter measured on a 150 vessels sample, fit ${y}={{p}}_{1}{x}+{{p}}_{2}$ with p_1_ = 3.7, p_2_ = −8.2, R^2^ = 0.70. (**D**) Dependency of maximum velocity with vessel’s diameter measured on a 150 vessels sample fit ${y}={{p}}_{1}{x}+{{p}}_{2}$ with p_1_ = 1.9, p_2_ = −6, R^2^ = 0.60.

As expected, Fig. 3A shows that the reconstruction of big vessels is faster than in capillaries. Indeed, microbubble supply should decrease as vessels get smaller and flow rate decreases. To highlight the time for reconstruction of single vessels, we isolated 150 vessels with sizes between 5 μm and 100 μm (Fig. 4A). For each vessel, we counted the total number of microbubbles passing through a given cross section in time (Fig. 4B). Typically, a vessel of 100 μm sees a dozen microbubbles every second whereas a vessel of 20 μm only sees one microbubble every 10 s. For smaller vessels, we expect to see at most one microbubble during the 4 minutes of the course of the experiment. For different size of vessels, we see that the passage of microbubbles in the vessels is overall linear with time, which is consistent given that the vascular concentration of microbubbles is constant. We can also estimate the flow rate’s behavior with the vessel sizes by plotting the total number of microbubbles detected in 240 s through the cross sections as a function of the vessels diameters (Fig. 4C). Indeed, according to [2], they should be proportional. The log-log plot exhibits a global power law for the microbubble flow with the diameter. The exponent is slightly lower than 4 which would be the Poiseuille power law [1]. On the same vessels, we can report the mean velocity as a function of the diameter (Fig. 4D). In this case the log-log plot shows an overall power law with an exponent close to 2. On Fig. 4C,D the trends follow the model we define but with poor determination coefficients. This confirms that choosing the diameter as the determinant is relevant for a first order estimation but hides a lot of finer effects.

**Figure 5:**
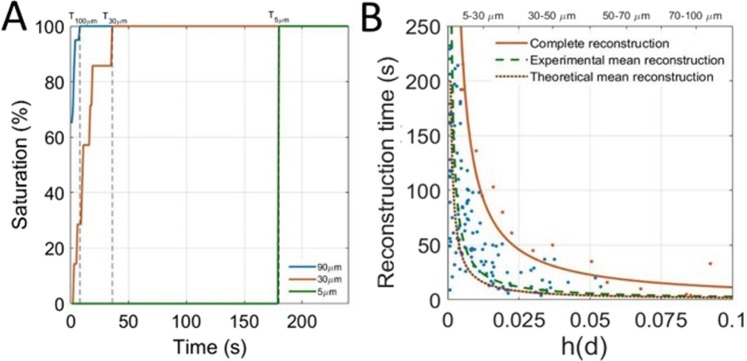
Determination of the influence of the acquisition time on vessel reconstruction. (**A**) Determination of reconstruction times for vessels of 90 *μm*, 30 *μm* and 5 *μm*. (**B**) Scatter plot of saturation times of vessels by size. Complete reconstruction curve (orange line)${y}={{p}}_{1}{{x}}^{-1}$ with p_1_ = 1.14 μm^−1^, R^2^ = 0.97, Experimental mean reconstruction curve (green dashed line)${y}={{p}}_{1}{{x}}^{-1}$ with p_1_ = 0.29 μm^−1^, R^2^ = 0.72. Theoretical mean reconstruction curve (brown dashed line)${y}={{p}}_{1}{{x}}^{-1}\,$with p_1_ = 0.2 μm^−1^.

To study the reconstruction rate of vessels according to their diameter, we consider the saturation of the cross section of the 150 selected vessels along time. The saturation curves in Fig. 5A also captures the time necessary for the vessels to be completely reconstructed (e.g. saturation reaches 100%). In Fig. 5B, a scatter plot represents the saturation times for each individualized vessels as a function of its diameter *h(d)* determined in [5]. The experimental mean reconstruction curve (Dashed brown line) is determined through an inverse linear regression on these points and represents the average reconstruction time for a given size of vessels. The model Equation [6] is used to predict a theoretical mean reconstruction curve (dashed green line) which seems to be in accordance with the experimental mean reconstruction curve. A family of vessels is reconstructed when all corresponding vessels are reconstructed. Therefore, the time to reconstruct a family of vessels is limited by vessels with the slowest reconstruction (singled out in orange). Inverse fitting on these points form the complete reconstruction curve (orange line) that set the required time for vessels of a given size will be reconstructed.

Vessels between 70 μm and 100 μm are reconstructed within 10 s, whereas vessels between 30 μm and 50 μm require at least 40 s of acquisition times. The reconstruction times increase drastically and it might take more than 10 minutes to fully populate all vessels below 25 μm. It is very likely that a fraction of very small vessels (<25 μm) are missing as they haven’t been explored by any microbubble. Such hypothesis is similar to the analysis of saturation curves in Fig. 3, that for 5 μm pixels, the 100% saturation is expected around 10 minutes.

### Influence of acquisition time on the reconstruction of velocity profiles

**Figure 6:**
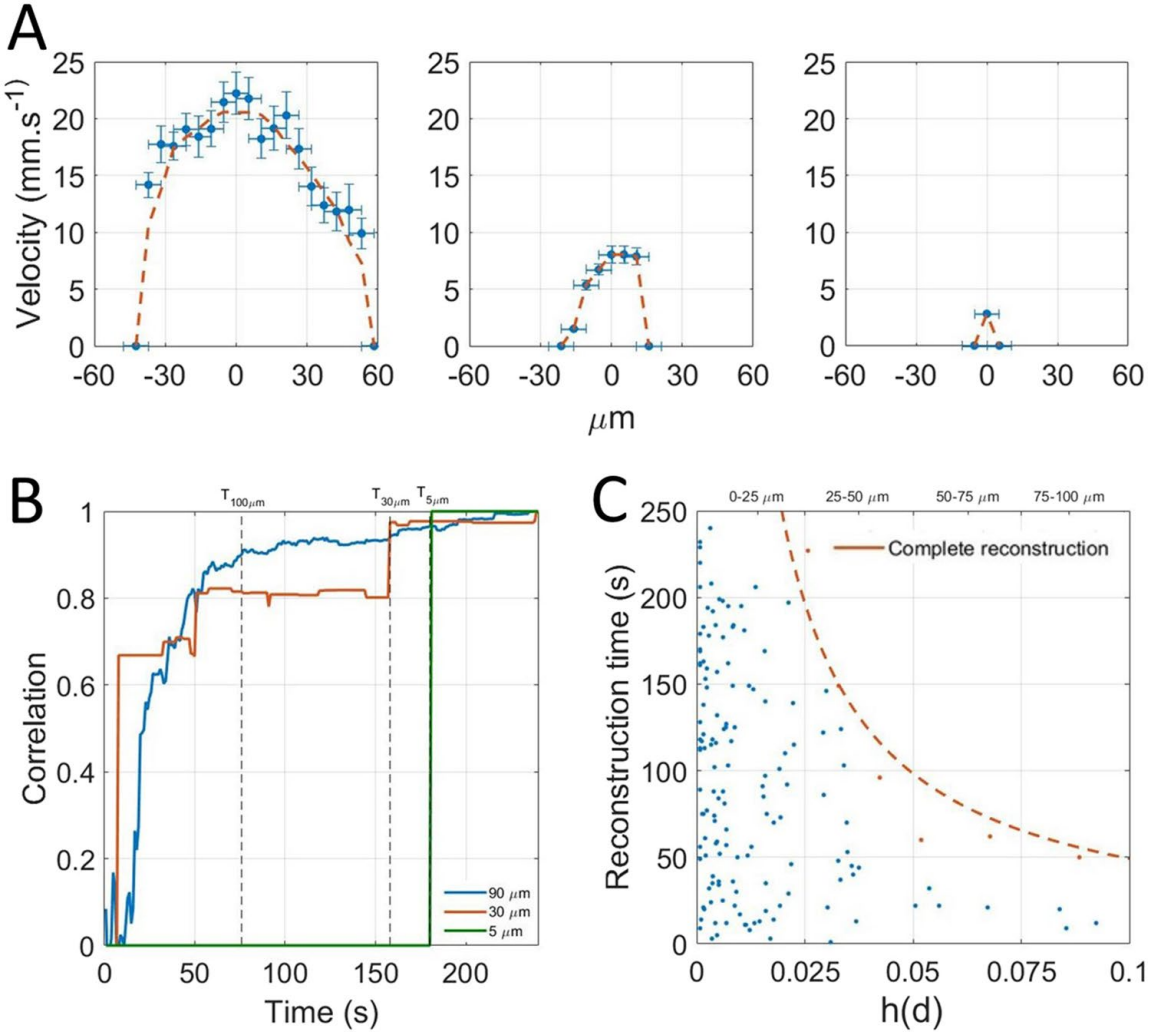
Determination of the influence of the acquisition time on velocity profiles reconstruction. (**A**) Velocity profiles for three vessels of 100 *μm*, 30 *μm* and 5 *μm*. (**B**) Correlation with complete profile to determine reconstruction time. (**C**) Scatter plot of reconstruction times of vessels by size. Complete reconstruction curve (orange line) ${y}={p}_{1}\frac{1}{{\boldsymbol{x}}}$ with p_1_ = 4.9 μm^−1^, R^2^ = 0.96.

If the detection of one microbubble is a necessary condition to reconstruct a pixel, it might not be sufficient to have a good contrast or to estimate velocities. Indeed, because of the absence of elevation resolution, all the microbubbles passing through a vessel can have different velocities inside a vessel because of their position in elevation and the orientation of the vessel. Therefore, averaging over several microbubbles is needed to converge on an underestimated but stable velocity profile. Nonetheless, to estimate reconstruction time for velocities, we measured a velocity profile along the cross sections (Fig. 6A). The correlation between the profiles reconstructed after different times with the profile obtained at the end provides a measure of how fast the profiles are reconstructed (Fig. 6B). The reconstruction time is set as the time to reach a correlation of 0.9. Similarly to Fig. 5B, the reconstruction times for the 150 vessels are reported as the blue points on the scatter plot on Fig. 6C. In Fig. 5B the slowest vessel to reconstruct are singled out (orange points) and the complete reconstruction curve (orange line) was calculated with an inverse fit.

Even though the precision on the measure of velocity of a single microbubble can be very fine, it is necessary to average the measure several times to converge on a stable velocity, mainly because of pulsatility, fast hemodynamic changes but most importantly because of poor elevation resolution. In the elevation dimension, it is impossible to distinguish a microbubble flowing on the side or in the center of a vessel. For a central pixel, the estimation of velocity can be greatly affected. Similarly to the determination of the complete reconstruction curve, a complete reconstruction curve for velocity profile can be established. Reconstruction times are 4 times higher than the simple density imaging. Therefore, one should be careful exploiting the velocity profiles as the central velocity is likely to be underestimated. Velocity in vessels between 70 μm and 100 μm are reconstructed within 50 s whereas vessels between 30 μm and 50 μm require at least 160 s of acquisition times. For the smallest vessels it would take several tens of minutes.

## Discussion

The goal of this study was to establish the link between the acquisition time and scale at which the microvasculature can be described by ULM. We assumed that, as vessels diminish in size, microbubble rate of passage diminishes as well. Through a simple blood flow model, we propose that the necessary acquisition time to reconstruct a vessel is inversely proportional to its blood flow, meaning that small vessels require longer acquisition times. For instance, we show that larger vessels of 100 micrometer are fully reconstructed within 10 seconds, but that detecting all the smallest vessels, would take minutes to tens of minutes for capillaries. The reconstruction of velocity profiles can take up to 5 times longer as compared to the microbubble distribution mapping, since it requires a greater number of microbubble tracks.

As it stands, ULM could provide images of the complete capillary network with a 5 μm accuracy in about ten minutes with total or partial removal of the skull. Compared to other existing techniques used to image the microvasculature, ULM offers a different set of advantages and drawbacks. For preclinical and clinical study of the vasculature, in depth imaging is only possible with a spatial resolution up to 100 μm with ultrafast Doppler^25^ or contrast MRI^26^. Using two photon microscopy, imaging of vessels can be done through a cranial window with a resolution below 1 μm but with a depth limited to under a millimeter^27^. ULM provide a compromise between optical microscopy and in depth imaging methods at the cost of several minutes of temporal resolution. As velocities can be accurately measured, it may be used as a quantitative tool to measure blood flow in depth and at the scale of individual vessels.

However, for accurate quantification, two question should be addressed. Firstly, how do microbubbles behave in the blood, especially in the smallest vessels? We hypothesized that microbubbles follow blood flow but the notion of blood flow becomes irrelevant when the size of the vessel approaches the size of microbubbles and red blood cells. Secondly, how does the lack resolution in elevation affect the ability of ULM to clearly quantify velocities and blood flow? The thickness of the image is still in the order of magnitude of λ and all vessels are projected in one plane. 3D ULM will provide resolution in all three direction, precise velocity estimations and registration for off plane motion. Nevertheless, as we increase the dimensionality, we foresee that the acquisition time will also be increased compared with a single plane reconstruction. We can adapt Equation [3] in [3′] and adapt [4] to determine the acquisition time [4′] for a 3D ULM acquisition.

3&x02032; $${N}_{3D}=\frac{{d}^{2}}{{{l}_{pix}}^{2}}$$

and

4&x02032; $${T}_{acq,3D}=\frac{1}{\frac{Q(d)}{{d}^{2}}{C}_{bubbles}{{l}_{pix}}^{2}}$$

Even if 3D ULM will ultimately be a more precise a quantitative way to perform ULM, most preclinical and clinical studies will first be conducted using 2D imaging. In both cases, the model we introduced can be helpful to design ULM acquisitions. We foresee that ULM could be a relevant imaging modality in two main situations. First, ULM is a unique imaging modality to study the microvasculature. We showed that in this situation, acquisition time is limited by low blood flow in the smallest vessels even with high microbubble concentrations. In this study, with a steady microbubbles infusion of 0.8 ml.kg^−1^ we expect complete capillary reconstruction in about ten minutes. With microbubbles concentrations within clinical recommendations (0.03 ml.kg^−1^), we expect the reconstruction time to be longer by roughly an order of magnitude. However, the clinical safety of SonoVue microbubbles has been evaluated for doses between 0.003 and 1 ml.kg^−1^ and concluded that even high doses of microbubbles appeared safe and well tolerated^21,22^. Therefore, for clinical applications, it could be possible to increase microbubble dose to reduce reconstruction time. However it could only be increased to the point where microbubbles stop being individualized, especially at lower imaging frequencies were pixel sizes are larger. This comes as a fundamental limit for capillary imaging where the acquisition time is necessarily of several minutes. Major methodology changes, for example the use of smaller yet more numerous microbubbles or activable nanodroplets^28^, may eventually populate the capillary bed faster and enable quicker reconstruction. Secondly, ULM would be relevant to image sub-millimeter vessels but deep into organs or through the skull. In bigger vessels, for microbubbles to be individualized, the concentration should be lowered, eventually extending the acquisition time. Thus, even with a lower emphasize on capillary structures and low blood flows, the acquisition time should remain relatively long as compared to conventional ultrasound Doppler.

## Conclusion

This study demonstrates that ULM performances in terms of spatial resolution come at the cost of extension of the acquisition time. As microbubbles behavior follows the physiology of the vasculature, we propose that image reconstruction in ULM is above all driven by blood flow. Microbubble concentration have to be adapted to the vessels under investigations, it should be lowered to image sub-millimeter vessels and increased for capillaries. In all cases, as the key to super resolution is having isolated microbubbles, it imposes a fundamental limitation to only see tens of different microbubbles in each frames. Therefore, acquisition time is bound to be of several minutes and even tens of minutes to image the capillary bed and the slowest vessels. Compared to the drawbacks of other comparable imaging modalities, several minutes of acquisition times doesn’t appear so limiting as ULM could provide a clear window to capillaries and deep vascular structures in animal and humans. A window that could help us understand and diagnose many diseases where the microvasculature plays an important role such as cancer, stroke, diabetes and arteriosclerosis.

## Data Availability

Raw data and image processing algorithms can be exchanged throughout a collaboration agreement. Processed data and analysis algorithms can be made available on demand.
